# Patient and Public Engagement in Integrated Knowledge Translation Research: Are we there yet?

**DOI:** 10.1186/s40900-019-0139-1

**Published:** 2019-02-12

**Authors:** Davina Banner, Marc Bains, Sandra Carroll, Damanpreet K Kandola, Danielle E Rolfe, Caroline Wong, Ian D. Graham

**Affiliations:** 1 School of Nursing, University of Northern British Columbia, 3333 University Way, Prince George, V2N4Z9 Canada; 2 Ottawa Hospital Research Institute, Ottawa, Canada; 3 HeartLife Foundation, Vancouver, Canada; 4 School of Nursing, Faculty of Health Sciences, McMaster University and Associate Scientist, the Population Health Research Institute, Hamilton, Canada; 5 School of Health Sciences, University of Northern British Columbia, Prince George, Canada; 6 School of Epidemiology and Public Health, University of Ottawa, Ottawa, Canada; 7 Centre of Excellence in Partnership with Patients and the Public, Montreal, Canada; 8 School of Epidemiology, Public Health and Preventative Medicine, University of Ottawa and Centre for Practice Changing Research, Ottawa Hospital Research Institute, Ottawa, Canada

**Keywords:** Integrated knowledge translation, Knowledge translation, Patient and public engagement, Patient-oriented research, Partnerships

## Abstract

**Plain English summary:**

There have been many attempts to improve how healthcare services are developed and delivered. Despite this, we know that there are many gaps and differences in practice and that these can lead to poor patient outcomes. In addition, there are also concerns that research is being undertaken that does not reflects the realities or needs of those using healthcare services, and that the use of research findings in practice is slow. As such, shared approaches to research, such as integrated knowledge translation, are being used.

Integrated knowledge translation (IKT) is a research approach that brings together researchers, along with other stakeholders that have knowledge about a particular healthcare issue. Stakeholders may include healthcare providers and policy-makers. More recently, there has been a growing awareness of the need to include patients and members of the public within research processes. These collaborative and patient-oriented research approaches are seen as a way to develop research that tackles ongoing gaps in practice and reflect the insights, needs and priorities of those most affected by health research outcomes. Despite great support, little is known about how these major research approaches are connected, or how they may bring about improvements in the development and use of research evidence. In this paper, we examine how IKT and patient engagement processes are linked, as well as exploring where differences exist. Through this, we highlight opportunities for greater patient engagement in IKT research and to identify areas that need to be understood further.

**Abstract:**

Healthcare organizations across the world are being increasingly challenged to develop and implement services that are evidence-based and bring about improvement in patient and health service outcomes. Despite an increasing emphasis upon evidence-based practice, large variations in practice remain and gaps pervade in the creation and application of knowledge that improves outcomes. More collaborative models of health research have emerged over recent years, including integrated knowledge translation (IKT), whereby partnerships with key knowledge users are developed to enhance the responsiveness and application of the findings. Likewise, the meaningful engagement of patients, in addition to the inclusion of patient-reported outcomes and priorities, has been hailed as another mechanism to improve the relevance, impact and efficiency of research.

Collectively, both IKT and patient engagement processes provide a vehicle to support research that can address health disparities and improve the delivery of effective and responsive healthcare services. However, the evidence to support their impact is limited and while these approaches are inextricably connected through their engagement focus, it is unclear how IKT and patient engagement processes are linked conceptually, theoretically, and practically. In this paper, we will begin to critically examine some of the linkages and tensions that exist between IKT and patient-engagement for research and will examine potential opportunities for IKT researchers as they navigate and enact meaningful partnerships with patients and the public.

Rapid population ageing, burgeoning rates of chronic disease, and worsening health outcomes are changing the global healthcare landscape [1–3]. These key problematic health issues, in concert with escalating healthcare costs and widening health disparities, are driving a renewed focus on the need for evidence-based healthcare services that bring about improvements in health system and patient outcomes [4, 5]. Despite this increasing emphasis, gaps in the creation and timely utilization of evidence in healthcare pervade and large variations in practice remain [6–10]. Over recent decades there has been an increasing recognition of the need for more engaged approaches to health research. The traditional passive models of knowledge creation and transfer are becoming replaced with greater transdisciplinary and inclusive research approaches that have evolved to reflect the complexities of healthcare environments, as well as the needs and preferences of patients and other knowledge users [11–14]. In Canada, this collaborative approach to research is commonly called Integrated knowledge translation (IKT).

IKT is a collaborative model of research that engages knowledge users, including decision-makers, healthcare providers, policy makers, patients, caregivers and members of the public, as partners for research [15]. Separate from end-of-grant knowledge translation (KT), IKT requires that knowledge users and researchers work collectively across the research process to identify key priorities, develop responsive research questions, interpret findings, and advance the application of research outcomes into practice [16]. While IKT is underpinned by distinct vocabulary, the underpinning principles are common to many other collaborative research approaches, such as engaged scholarship [17–19], community-based participatory research [20], and mode 2 research [21], each reflecting a commitment to co-production, mutual decision-making and knowledge exchange [22, 23]. Despite these disciplinary variations, IKT has garnered an increasing international presence over recent decades [21–25].

In concert with a growing emphasis on such integrated approaches, the meaningful engagement of patients and the public has been hailed as a key mechanism to improve the relevance, impact and efficiency of research [26–32]. This engagement, sometimes referred to as patient and public involvement, service user involvement, or citizen engagement, represents an important cultural shift as evidenced by the integration of major national research frameworks and strategies, including the Canadian Institutes of Health Research (CIHR) Strategy for Patient-Oriented Research (SPOR) [33, 34], National Institute for Health Research (NIHR) INVOLVE in the United Kingdom (UK) [35], and Patient-Centred Outcomes Research Institute (PCORI) in the United States of America [36]. Moreover, the World Health Organization (2006) recognizes the importance of patient and public engagement across all health sectors, including policy and governance [26]. In essence, these initiatives and strategies are underpinned by a common ethos that research should be “*carried out ‘with’ or ‘by’ members of the public rather than ‘to’, ‘about’ or ‘for’ them*” (p.6) [37] and responds to the movement of ‘*nothing without us’* [38, 39] which calls for full participation in decision-making around healthcare, policy and research. Within these international frameworks are varied taxonomies and language, which can give rise to some confusion. In the Canadian SPOR framework, the terms patient engagement and patient-oriented research (POR) are typically used, with ‘*patient*’ being considered an all-encompassing and inclusive term to include those with lived experience of a health concern, as well as caregivers, family members, friends and members of the public [33, 34]. For clarity, we will use the term patient engagement in this commentary to reflect these varied positions.

Through a rapidly evolving mandate to engage with patients and members of the public in research, there has been a changing discourse around the nature of evidence and a growing desire among patients and the public to contribute to research. This has led to an increased recognition of the need for high quality data that also incorporates the experiential knowledge of those that are impacted by the research [37, 40]. As a concept and practice, McKevitt (2013) argues that patient and public engagement in research has rapidly become “*rhetorically and structurally embedded in the field of publicly funded medical research*” (p115) [37]. Further, this engagement imperative is seen as a means of promoting greater accountability, authenticity, transparency, and trust in the scientific endeavour, while fostering more democratic and socially responsible practices that challenge traditional academic elitism and privileged knowledge [20, 41–44].

There has been a recent surge in activities around patient and public engagement and a rapidly growing body of literature. However, there is great variation in how patients are engaged in research and the evidence-base to support its impact is highly fragmented [30, 31]. Furthermore, there is little consensus about how patient engagement is conceptually or theoretically linked to other research approaches and frameworks. The aim of this paper is to begin to explore patient engagement within the context of IKT research. Through this we are able to take a critical look at two dominant approaches in contemporary health research, making explicit how the principles, practices and outcomes of patient engagement and IKT are complementary and where tensions exist. By beginning to understand this further, it is possible that IKT research teams may be better able to develop and undertake research that fosters the meaningful engagement of patients as knowledge users, and further incorporates outcomes of interest to patients and the public. Likewise, delving into these complementary and overlapping processes responds to a current gap in the literature and may fuel the advancement of science for both IKT and patient engagement by identifying areas that require further research.

## IKT research: Engaging knowledge users to drive improvements in patient care

Over recent years, a widespread transition in health research has occurred that has resulted in a move away from more traditional knowledge transfer approaches to the introduction of more complex, contextualized, and engaged modes of creating and implementing evidence [12]. This occurred in part due a failure to address ongoing variations in practice, but also as a means of bridging ‘know-do’ gaps to develop effective and efficient evidence-based healthcare services [45–48]. For example, the creation and integration of evidence to improve clinical outcomes is challenging and recent studies highlight that only 60% of clinical decisions are rooted in an appropriate evidence base, with fewer than half of patients receiving the correct level of care and 25% receiving unnecessary or potentially harmful care [8–10]. Also, waste and inefficiencies in health research, along with systems that incentivize quantity and competition as opposed to collaboration and quality, continue to be a significant and costly issue, accounting for over US$200 billion in wasted research revenue in 2010 [49, 50]. Furthermore, there are ongoing concerns about the failure of researchers to develop studies that reflect the ‘*real world*’ nature of clinical practice, further contributing to lengthy delays in its translation and utilization. Coined as the ‘*death valleys’*, there is increasing pressure for decision-makers, healthcare providers, patients, and researchers to bridge these ‘*know-do’* gaps and develop effective and efficient evidence-based healthcare services [5, 12, 15]. Part of this transition has emerged in response to the increasing awareness that evidence in isolation is not adequate to drive health system change, rather, the broader perspectives of knowledge users, including patients and the public, are needed in order to generate and translate research that is actionable and impactful [22, 51].

In the global context, there are a plethora of concepts, models, theories, and terminology that contribute to this engagement mandate, including engaged scholarship and community-based participatory research [17, 18, 20], each with differing epistemological orientations and disciplinary foundations [22, 51]. In Canada, this shift to more engaged forms of research is seen through an increasing focus upon IKT within major health research funding programs and the growing expectation that researchers will work collaboratively to accelerate knowledge creation and translation to improve health outcomes [52–54].

Founded on the principles of knowledge-to-action, IKT fosters meaningful connections and partnerships to optimize the relevance and impact of the research and facilitate its application into practice [25, 45, 55, 56]. Through the IKT process, teams of researchers and knowledge users work collaboratively to engage in research that responds to the contextual, cultural, and social realities of the healthcare setting, mitigate key logistical and translational barriers, and develop outcomes of interest to those delivering and experiencing care [45, 57]. As such, IKT processes transcends common disciplinary boundaries, fostering pleuralistic and responsive research practices within cycles of knowledge creation and practice-oriented action [58, 59]. As Kothari and colleagues (2017) highlight, “*this new way of working suggests that the synergies derived from the collaboration will result in better science; more relevant and actionable research findings; increased use of the findings in policy or practice; and mutual learning*” (p.299) [25].

Over recent years, there has been an explosion in IKT research, as well as commentaries and syntheses that espouse the potential application and value of this collaborative research approach [25, 56, 59–64]. This has included a scoping review examining the use of IKT strategies in healthcare [65], literature examining the impact of research partnerships [14, 66, 67], as well as programmatic evaluations and practice innovations [52, 68]. For example, Harrison et al. (2005) undertook a before-after implementation study examining a community approach to leg ulcer management. As part of this, an interdisciplinary team of decision-makers, healthcare providers, and researchers collaborated to evaluate the existing models of care and introduce a nurse-led community leg ulcer service. Following implementation of this new care model, statistically significant increases in the uptake of guideline-based compression therapy were observed, in addition to improved healing rates and reduced healthcare costs [69]. Overall, teams adopting IKT approaches commonly report that the research resulted in improvements in outcomes and health systems improvement [52].

Despite the growing acceptance of IKT approaches, evidence to support its use is somewhat limited [63]. To date, many of the published studies fail to adequately report on the nature and scope of knowledge user engagement or evaluate its impacts more broadly [64]. Thus, a lack of consistent and distinguishable evaluation processes can make it challenging to assess the magnitude of its impact upon the research process or long-term outcomes. Further, other studies have highlighted that resource and time constraints, along with a lack of attention to power and politics, can be major barriers to undertaking IKT, contributing to a lack of uptake on occasions [24, 70]. Even in the face of these challenges, there is growing investment and support for IKT and engaged forms of research. This is based on the promise of more relevant health research and the ability to bridge different disciplinary and experiential knowledge to improve outcomes [11–14, 71, 72]. However, while patients, families and members of the public are considered valid knowledge users within the IKT paradigm, these stakeholders are not systematically engaged. Since patient engagement strategies represent a dominant discourse in contemporary health research, there is an opportunity to take a renewed focus on IKT by exploring how and where patients are engaged in IKT and to examine its impact.

## Patient engagement in Health Research: A new frontier

Patient engagement activities are centred around the need to meaningfully engage individuals and communities in initiatives to advance healthcare [31]. While the practice of involving patients and members of the public in research is well established across many disciplines [4, 5, 58], the strategic and widespread engagement of patients to guide research, including its focus and outcomes, represents a new frontier in contemporary health research [6, 7]. This is particularly true in Canada, whereby the patient-oriented research initiative is still a relatively recent addition to the health research landscape [33, 34]. Researchers are increasingly expected to systematically engage patients in research, with the purpose of driving the development of studies that reflect the needs and priorities of patients, and to optimize the uptake of the evidence into practice [33]. In response to this, a plethora of strategies and frameworks have emerged as a means of fostering the authentic engagement of patients in research, alongside larger scale initiatives and structures that have influenced the prioritization and funding of research more broadly [34, 36]. In Canada, the CIHR SPOR initiative highlights that researchers must create a ‘*strong foundation*’ for meaningful patient engagement and facilitate this engagement across the continuum of research, in order to “*build a sustainable, accessible and equitable health care system and bring positive changes in the health of people living in Canada*” [33] pg. 4).

Early examples of successful engagement can be found within the Human Immunodeficiency Virus (HIV) and arthritis literature [73–78], whereby patient activism and agency generated critical influence upon the operationalization of healthcare services and the focus and uptake of research. For example, in the case of rheumatoid and psoriatic arthritis, patient activism has led to a ‘reimagining’ of the healthcare journey among health researchers. Through this, patients have contributed pivotal insights on the wider impacts of their disease, as well participating within broader health services and research priority setting and evaluation [79, 80]. More recently, there has been an expansion in the literature identifying the benefits of patient and public engagement in research, including improvements in the recruitment of participants and the uptake of research findings [81, 82]. For example, Ennis and Wykes (2013) undertook an analysis of 374 studies undertaken as part of the Mental Health Research Network in the UK. In their analysis, levels of involvement, study complexity and recruitment practices were examined across groups of studies. The authors identified that while engagement was varied, teams with higher levels of patient engagement were more likely to have achieved their recruitment targets [81]. Overall, the benefits of combining the *experiential* insights of patients, along with the empirical and theoretical knowledge of researchers and other knowledge users, has been seen as providing the optimal strategy for improvements in healthcare and research [58, 83–85].

### The nature and scope of engagement

Numerous models and frameworks exist that seek to conceptualize the patient engagement process [86–88]. Within these, there are common principles that speak to the nature of engagement, these include the need for: 1) authentic and sustained engagement across the research continuum and beyond, 2) clarity in the roles and expectations of all parties engaged in the research, 3) mutual trust and respect, 4) commitment to co-learning and co-production, and 5) access to the appropriate resources, supports and training. Likewise, there are a growing number of typologies that seek to delineate the scope of engagement [89–93]. For example, the International Association for Public Participation (IAP2) Patient Participation Spectrum presents a tangible and practical scale through which to conceptualize and evaluate patient engagement in health research [92]. Such typologies highlight the continuum of engagement activities, including the involvement of patient to provide experiential insights, to full collaborative partnerships that foster co-creation and co-production in research, and empowerment and leadership in decision-making. Overall, proponents of patient engagement highlight that these highly inclusive research strategies offer an opportunity to increase the responsiveness and translation of research.

### Gaps, uncertainties, and variations

Recent bureaucratic and cultural shifts have seen a driving mandate to foster the engagement of patients and members of the public in health research [94, 95]. Despite a growing body of literature to support patient engagement, gaps, uncertainties, and variations in the reporting and evaluation of engagement activities exist [30, 31, 96] and there are continued calls for more robust evaluation of engagement processes, along with studies that systematically examine its impact [32, 97–101]. For example, Brett and colleagues (2014) undertook a systematic review of 66 papers exploring patient and public participation in research. The analysis of the literature identified that there was a growing evidence to support engagement across the research process, including the conceptualization of the research question and study recruitment practices. However, they identify that the overall evidence-base was weak and more robust studies that explore the impact of patient engagement are urgently needed [30]. Likewise, a systematic review by Mockford et al. (2012) highlighted that while there was growing evidence of health service improvements as a result of the engagement of patients and the public, including contributing to a greater awareness of patient priorities and improved KT, a failure to adequate report the process and nature of engagement, in addition to a lack of measurement of the impact or cost benefits, made it challenging to assess the broader evidence-base [31]. Finally, Domecq and colleagues (2014) undertook a systematic review of patient engagement, identifying that while patient engagement was both feasible and led to potential improvements in recruitment, funding success, and the identification of outcomes, engagement was typically isolated to the early stages of study development, with few studies reporting engagement during the implementation and translation phases of the research process [32]. Similar findings were also found by Concannon et al. (2012) [88] in their systematic review examining stakeholder engagement in comparative effectiveness and patient-centered outcomes research.

Further ambiguities exist relating to the potential contributions of patient engagement. Such obscurity is largely linked to a lack of clarity about engagement processes, fears of tokenism, variations in language, disciplinary norms, and skepticism relating to the potential impact of engagement in light of a limited evidence base [102–104]. For instance, concerns and uncertainties relating to patient engagement were raised in a recent study by Carroll et al. (2017) that explored the perspectives of cardiovascular research scientists. Participants in this study indicated uncertainty with respect to how patients are engaged, concerns around the potential knowledge divide between patients and researchers, and apprehension about the increased cost and time required to engage patients, with little promise of return on outcomes and evidence uptake [105]. Likewise, concerns that engagement may be superficial and may fail to capture the perspectives of those most impacted by the research have also been raised [32, 105–108].

Finally, inconsistencies in the underpinning definitions and reporting of patient engagement activities are contributing to variations in how patient engagement is understood and enacted [103, 109]. This is what Forbat and colleagues term as “*conceptual muddling*” and is further complicating attempts to measure and evaluate its impact (p. 2553) [91]. For example, in Gallivan et al.’s (2012) mixed methods scope-defining study, stakeholders identified a lack of clarity around the concept of engagement and the associated terminology. Within the 23 articles reviewed as part of this literature review and focus group study, 15 conceptual terms related to patient engagement were identified, including patient involvement and public participation, with few studies specifically using the term patient engagement or delineated the purpose or goal of engagement [103].

Despite the presence of such uncertainties and a fragile evidence base, patient engagement has become widely accepted. Even in light of these gaps, it would be hard to disagree that the engagement of patients represents an important democratic and ethical practice in contemporary health research and provides an opportunity to address some of the complex challenges related to the creation of responsive and actionable healthcare evidence [110]. Further dialogue and inquiry is needed to address these gaps and to examine how patient engagement is enacted and positioned within the context of established research methodologies, practices, and theories. Here, we begin to explore patient engagement within the context of IKT research and seek to identify how these dominant approaches overlap and where opportunities exist to further expand patient engagement in IKT programs and research.

## Patient engagement in IKT research: Are we there yet?

Efforts to support the engagement of knowledge users and patients in research represents a relatively new, yet widely acknowledged, mechanism for promoting greater accountability, authenticity, transparency and trust in the scientific endeavor [7, 23, 24, 111]. Furthermore, others have argued that such engagement offers further political gain by fostering more democratic and socially responsible scientific practices, increasing the legitimacy of experiential knowledge, and responding to the ethical and moral imperative that those impacted by the research should have a role in its construction and execution [32, 112]. If researchers are to respond to these growing expectations, as manifested through the proliferation and tailoring of research funding competitions aimed at fostering greater engagement, researchers need to establish robust engagement and partnership practices.

Ongoing gaps in the IKT and patient engagement evidence-base, as well as well documented complexities in undertaking research processes that are both meaningful and impactful, are pervasive. For example, Gagliardi and colleagues (2015) undertook a scoping review of 13 healthcare studies that used and evaluated IKT approaches [65]. The authors highlighted that IKT practices are highly variable and are often poorly reported and evaluated. While not the purpose of this review, we re-examined the captured literature to explore where and how patients or community members were engaged. From this, only three research teams had indicated that patients were engaged within the research [61, 62, 113], of which these were largely those who held a formal role within community organizations, with only one article provided a detailed overview of the nature and scope of the engagement activities [61]. While we recognize that this review is by no means exhaustive and has not have assessed all of the IKT literature available, we argue that the healthcare focus of this scoping review would be positioned to capture research that would be of most concern to patients and would thus warrant the potential engagement of patients as knowledge users. Thus, we contend that opportunities exist to foster the more meaningful and widespread engagement of patients as knowledge users in IKT teams. To further examine the opportunities and tensions that exist for patient engagement within IKT research, we will now explore the related conceptual and theoretical considerations.

## IKT and patient engagement: Conceptual and theoretical considerations

While patient engagement may have gained a more “*pragmatic accommodation*” in health research today (pg. 595) [44], there has been little research that has examined this within the context of existing methodologies and research approaches. Rolfe et al. (2018) contend that despite the abundance of patient engagement frameworks and tools, the examination of patient engagement within the context of existing research methodologies and approaches remains poorly understood and can contribute to practical challenges and ethical dilemmas [114]. Furthermore, as the research around patient engagement is expanding, concerns about a lack of authenticity and tokenism are continuing to emerge [98, 107]. For example, there is an emerging body of literature identifying the importance of patient engagement in the context of clinical trials, although the evaluation of the process and impact of engagement frequently remains unexamined and under-reported [32, 115–117]. Bagley et al. (2016) further identifies the fragile balance between fostering patient engagement and input in such designs and the need to safeguard scientific methods and rigor [87]. Consequently, greater attention is needed to explore the conceptual, theoretical, and methodological impacts of such engagement within the context of the wider research process. Here, we examine opportunities for patient engagement as it relates to the purpose, process and outcomes of IKT research.

### Purpose

Both IKT and patient engagement comprise activities and strategies that seek to foster engagement across the research process, typically such engagement is undertaken for the purpose of enhancing the relevance, responsiveness, and applicability of the research or healthcare intervention [15]. However, it could be argued that distinct differences and tensions may exist in how these engagement process are understood within the context of IKT research and patient-oriented research more broadly.

The IKT approach was explicitly established to create a platform for the engagement of a broad range of knowledge users, including patients and members of the public. Irrespective of the designs and methods used, research that is underpinned and framed by the IKT approach values the perspectives of those who are impacted by the research, or those that may use the research findings to bring about wider system change [15]. Thus, patients are well positioned to contribute meaningfully within IKT teams.

In IKT research, knowledge users are engaged as partners in the research to provide distinct expertise pertaining to the context and focus of the research. Knowledge users are typically those that have the ability to bring about or influence change and implement the resulting outcomes or recommendations [15]; while researchers are engaged to provide methodological and scientific expertise [25]. In the literature to date, knowledge users that have been engaged in IKT research are most commonly those that provide frontline services, inform policy, or have decision-making authority within a healthcare organization. Thus, few teams engage patients in a systematic way. However, patients, members of the public, and representatives of external organizations and patient advocacy groups may also be engaged as knowledge users to provide critical insights into a given lived experience, as well as promoting broader linkages with a given community. For example, partnerships with patient advocacy groups may be particularly important if the population of interest may experience multiple barriers to engagement, such as children, individuals with rare diseases, or those facing social disadvantage [82].

Within IKT research, the engagement of knowledge users is developed to provide diverse perspectives of a given research problem and to reflect the unique context within which the research takes place. In contrast, some patient-oriented research activities may have a specific agenda that is focused principally upon the analysis and articulation of patient needs, priorities and values [118]. Thus, the focus here shifts away from the broader context of the research, to the purpose of identifying and acting upon patient needs and priorities specifically. However, while attempting to draw upon these differences and similarities, we too recognize that the nature and purpose of engagement within both IKT and patient-oriented research has not been systematically studied and that engagement modes and practices may morph and shift as the research process unfolds. Further research that seeks to delineate and evaluate patient engagement in IKT research may yield more nuanced understandings of these processes.

### Partnerships

Partnerships are the bedrock of IKT research and typically evolve organically and strategically with the purpose of driving the focus of the research, augmenting its execution, and fostering widespread uptake in practice. Underpinning these partnerships is the need for researchers and knowledge users to work collectively and synergistically to address a common concern or issue [15, 56]. This approach, however, makes the assumption that all team members have equal ability to influence the research process and foster healthcare change.

Patients may become engaged in research to contribute a unique experience of illness or provide broader insights into the needs and priorities of a patient population, but patients may likely lack substantial decision-making power beyond this. Patient partners can be disadvantaged by a lack of clarity around the language and process of research, as well as being potentially the only unpaid team member within a broader collaboration of professionals and researchers. IKT approaches are conceptually oriented to address these imbalances, through a commitment to co-leadership and co-production, yet modes of practice must be established that seek to move beyond these theoretical positions to generate collaborative decision-making mechanisms to address the common purpose of the research. This should include having a clear understanding of the contribution and expertise of each member, leadership support for collaboration, as well as promoting effective strategies to allow for inclusive research approaches and outcomes [71].

In contrast, patient engagement may be understood as occurring across a wider continuum. For example, the IAP2 Spectrum of Patient Engagement determines the level of engagement that patient may assume within healthcare and research, and further delineates the varied goals and outcomes that may characterize each level [92]. Patient engagement may occur as a means of providing indirect support and may span simply being informed of the research, to being consulted or involved as an external stakeholder. In addition, more direct forms of engagement may occur, where patients may collaborate with a team or become empowered to direct or lead a given component of the research. It is these latter forms of engagement that would be considered most aligned with the epistemological and theoretical position of IKT research (see Table 1). However, IKT and patient engagement are not rigid or static processes and the partnerships that underpin both endeavors can be inherently changeable, complex, and non-linear [70]. Such idealism around partnerships may be tenuous, as IKT researchers often find themselves at the mercy of the ebbs and flows of the healthcare context or needing to respond to rapidly changing personnel and organizational foci [67]. Likewise, teams engaged in patient-oriented research may be similarly reliant upon the ability of patient partners to participate across the research process and may need flexibility to adapt activities in response to a patient partners’ changeable health status or expectations relating to the pace and timing of the research. This may prove challenging in the face of inflexible funding cycles and the requirements for rigid research protocols. Thus, the nature and scope of partnerships may change rapidly across the research process within both IKT and patient-oriented research and may span multiple levels of engagement as a result.

**Table 1:** Patient engagement and IKT goals and outcomes

IAP2 Spectrum^a^	INFORM	CONSULT	INVOLVE	COLLABORATE	EMPOWER
Patient Engagement Goals and Outcomes	Providing or sharing information with community and patient groups as a means of increasing awareness of a healthcare or research issue.	Garnering public feedback on research activities or outcomes. This may be undertaken to seek clarification or direction around a given issue.	Patients are engaged to provide insights to guide the decision-making within the research process. This may be isolated to key stages of the research or on an ongoing basis.	Patients and members of the public are engaged as members of the research team and contribute to shared decision-making across the research process.	Patients and members of the public provide direction and leadership about a given research endeavor.
Integrated KT Goals and Outcomes	IKT research team may tailor and share messages with community and patient groups, this in isolation would not be considered IKT research as patients or community members have not had the opportunity to engage in the wider decision-making.	IKT research teams may consult with members of the public during the research process as a means gaining input about the research process or outcomes. This activity would not be considered an IKT research process but may be considered an outcome of the IKT process.	IKT research teams may involve patients and members of the public to solicit input around decision-making, however, without full collaboration and decision-making authority, this type of engagement would not be considered IKT research.	IKT as a collaborative model of research fosters partnerships between knowledge users and researchers. When meaningful collaboration and shared decision-making occurs across the research process, this would be considered IKT research.	Within IKT research, team members contribute to the collaborative research processes, within which a patient may lead or be responsible for a specific element of the research. However, where patient leadership occurs independent of the broader team, this would be not considered IKT research.
Examples	Plain language summaries, publicly accessible reports, or social media messages.	Deliberative dialogue, town hall meetings and policy consultations.	Patient advisory councils or stakeholder priority setting activities.	Patient engaged as research co-lead or members of the research team.	Patient groups or members of the public voting about research priorities, or directing and leading research activities.

Ref: ^a^IAP2 Public Participation Spectrum https://iap2canada.ca/Resources/Documents/0702-Foundations-Spectrum-MW-rev2%20(1).pdf

### Representation, diversity and legitimacy

When engaging patients as partners in research, teams must consider their expectations about the varied contribution and perspectives that patients bring. Concerns about representativeness have been raised previously, with the idea that an individual patient may act as an ‘authority figure’ or sole representative of a patient population as being problematic and raising a number of practical and theoretical concerns [32, 105]. However, these concerns are largely unfounded and reflect the dominance of a narrow scientific model through which experiential knowledge is typically devalued or rejected [40]. Thus, teams must work to validate the experiential insights of the patient partner and accept this for its ‘intrinsic value’ [96, 119].

Concerns about diversity within both IKT and patient-oriented research have been raised, and there are calls to establish greater efforts to engage community members that may face multiple and intersecting barriers to engagement [120–123]. As described by Rolfe and colleagues, there are great divides and variations across most patient populations, particularly with respect to key demographic, cultural, ethnic, and social factors [114]. Furthermore, the notion of *who* constitutes a patient is varied and ranges from individuals that have personal experience of a health condition, to caregivers, family members, and friends, as well as members of the public and community organizations [33–36, 84]. A common criticism waged at the patient engagement movement is the potential to engage only the ‘*usual suspects’*, thus potentially privileging the voices of some, while possibly contributing to the ongoing marginalization of others [32, 70]. Without careful attention to the decision-making around patient engagement, it is possible that participation could be disingenuous and may continue to foster power imbalances. Incorporating an intersectionality lens may provide a means of addressing some of these concerns [108]. In both IKT and patient-oriented research, the purpose and nature of engagement should be identified clearly and steps taken to facilitate an inclusive approach that promotes co-production. For example, Cooke, Langley, Wolstenholme and Hampshaw (2017) highlighted the need to adopt methodologies and approaches that address the power and politics within teams and to incorporate creative activities and techniques to enable effective knowledge exchange and co-production [71]. Further research to examine and evaluate the impacts of power within both IKT and patient-oriented research is urgently needed.

### Process

Both IKT and patient engagement practices are founded upon the requirement for authentic and meaningful partnerships and a commitment to co-production [15, 22]. As previously highlighted, knowledge user and researcher relationships may arise in response to a common interest in a given healthcare population or setting. In essence, team members may be already working together or embedded within a specific healthcare system, which in turn positions them well to engage in a process of collaboration and shared decision-making. In contrast, the engagement of patients within an IKT team may not occur as organically, yet it is still intended that this partnership will directly contribute to the overall team process and decision-making. By examining and setting the tone for collective decision-making and participation, researchers can attempt to neutralize power imbalances and promote a more harmonious process of partnership [112–117, 119, 120]. This concern is by no way limited to that of patient engagement or IKT research, and discussions around promoting effective engagement have been an enduring topic of debate among many research disciplines, including in anthropology, community-based research approaches, critical feminism, ethnography, and sociology. While these established approaches have a clear epistemological, methodological, and theoretical positioning with respect for power and diversity, this is still emerging in the context of IKT and patient-oriented research.

While many research teams may be considered collaborative in nature, all too often the decision-making may be limited to a smaller group of researchers, particularly during the initial conceptualization stage. One may argue that this may be unavoidable in some circumstances, given current funding structures and models that may prohibit early engagement or may fail to provide the sufficient financial resources needed to sustain effective engagement. Consideration of the time, resources, and supports for such engaged approaches is needed if conditions for meaningful engagement are to be established. Since partnerships take time to develop, there is a need to work collaboratively early in the research process to actively establish mutuality, trust and reciprocity and address potential barriers including language and knowledge barriers, lack of resources and supports, and limited training opportunities [20, 86, 124–128]. Teams may therefore be charged with undertaking sequential and staged funding applications to support these processes, which in turn may potentially contribute to time increases and losses in efficiency.

### Outputs

Despite growing enthusiasm, the contributions and successes of patient engagement and IKT research are yet to be fully realized. While evidence to support these modes of research is beginning to emerge [73–80, 116, 129], efforts to systematically evaluate these have not been forthcoming [32, 101, 119]. Further research is needed that captures patterns of working, including the nature, duration and process of partnerships, as well as analyses of cost, resource use, time, and explicit impacts. Since both IKT and patient-oriented research seek to improve the relevance and timely application of research, robust evaluations and analyses of these processes may yield greater clarity in its use, as well as evidence to support or refute the allocated financial and structural investments [101, 118, 130–135]. This may further promote greater accountability and transparency in publicly funded research and provide an avenue to foster best practices in engagement.

These evidence deficits represent a significant gap in the contemporary literature and an opportunity exists for IKT researchers to continue to adapt and hone their engagement methods. This in turn may foster more sustained and productive engagement strategies and optimize their application to improve health system and patient outcomes more broadly. A growing number of frameworks, tools and toolkits to evaluate and report patient engagement are emerging in the contemporary literature [132, 136–138]. For example, Staniszewska and colleagues (2017) recently developed the revised GRIPP2 checklists, including a shortened version (GRIPP2-SF) oriented to reporting the engagement of patients, and a long form version (GRIPP2-LF) for studies whereby patient engagement and involvement is the principal focus. These international evidence-based guidelines were developed through consensus building methods and aim optimize the reporting of patient and public involvement in research [132]. Given the overlap between IKT and patient engagement, these emerging patient engagement frameworks and tools may provide an opportunity for greater consistency in the evaluation and reporting of IKT partnerships.

## Where can we go next?

IKT structures and process are largely synergistic to the strategies promoted through contemporary patient engagement initiatives. As IKT researchers seek to engage patients in a collaboratively and meaningful way, it is essential that teams pay attention to the development and enactment of these partnership and engage in research that allows for the explication and evaluation of the process. First, the IKT scientists and teams can begin to systematically explore the development of these partnerships and how effective working practices are established and experienced. In doing this, teams can work collaboratively to critically evaluate how participation and partnership develop and how this impacts upon decision-making, priority setting, and research outputs. Second, IKT teams should examine how the engagement of patients, and other knowledge users, impacts the focus and mandate of the research and to what extent the inclusion of knowledge users addresses the barriers and enablers to change in the healthcare setting. Third, IKT teams should be cognizant of how patient needs, priorities, and values are communicated and explored within research outputs. Attention to the nature of participation and dialogue around diversity and power will add to our understanding of the potential contributions of IKT and patient engagement. This may include exploring how issues relating to power are addressed and understood within teams. Finally, there is a need to critically examine episodes of discordance, as well as synergy, in the engagement process. This can give rise to a greater understanding of unexpected or unintended impacts. Potential research questions and patient engagement activities for IKT teams across the knowledge-to-action cycles are presented in Tables 2 and 3 respectively.

**Table 2:** Some research questions for IKT research teams around patient engagement

• How does patient engagement contribute to the process and outcomes of IKT research?	
• How can patient engagement in IKT research be understood and measured?	
• How can patient perspectives and partnerships impact upon the uptake of evidence-based healthcare practices, services or policies?	
• How can patient perspectives, priorities and values be examined and communicated within IKT research?	
• How can an intersectionality lens contribute to patient-oriented research and IKT?	
• What team characteristics foster optimal patient engagement in IKT research?	
• What are the unforeseen or unintended impacts of patient engagement on IKT research and implementation?

**Table 3:** Patient engagement across the IKT research process

KNOWLEDGE-TO-ACTION CYCLE	ACTIVITIES/OUTCOMES	POTENTIAL CONTRIBUTIONS OF PATIENTS WITHIN IKT RESEARCH TEAMS
Knowledge creation	Knowledge inquiry	• Identification of patient experiences, needs, priorities and values • Conceptualization of the research problem from a patient perspective • Co-creation of responsive research questions • Potential to contribute to the collection, analysis and interpretation of primary research data with the appropriate skill set (e.g. patient partners that have participated in formal research training).
	Knowledge synthesis	• Identification of patient experiences, needs, priorities and values • Facilitating opportunities for the co-creation of responsive questions for knowledge synthesis activities • Potential to participate in the collection, analysis and interpretation of research literature from a patient perspective. • Identification of relevant literature or resources for inclusion in the knowledge synthesis, such as grey literature and patient education resources.
	Knowledge tools	• Identification of patient experiences, needs, priorities and values to inform the development and testing of tools • Participate in the co-creation of responsive knowledge tools, such as patient decision-aids and KT tools.
Action Cycle	Identify knowledge gap	• Share lived experience of an illness or healthcare interaction • Create and foster linkages with a patient population or community • Identify knowledge gaps and barriers from the perspectives of patients • Engage in consensus approaches to determine patient priorities, such as a Delphi technique or Deliberative Dialogue
	Adapt knowledge	• Identify patient experiences, needs, priorities and values • Provide contextual insights and perspectives of health service users •Identify contextual factors that may impact the adaption and implementation of evidence
	Assess barriers and facilitators	• Share lived experience of an illness or healthcare interaction • Assist in the assessment and evaluation of barriers and facilitators within a healthcare context or the specific needs of a patient population • Identify potential ways to access a patient community to assess barriers and facilitators
	Select, tailor and implement interventions	• Participate in the prioritization of potential interventions or implementation strategies • Participate in the development of tailored and responsive healthcare interventions • Identify important research and evaluation questions relevant to implementation • Provide leadership or a lay perspective on the development of patient-centred healthcare interventions and tools
	Monitor knowledge user	• Identify and inform the development and implementation of evaluate tools and techniques • Assess the uptake or patient-oriented interventions or tools • Provide a connection with patient populations impacted by the healthcare issue and research
	Evaluate outcomes	• Provide input in to the development and implementation of evaluation activities and tools • Develop evaluation tools oriented for use by patients and the public
	Sustain knowledge use	• Lead or develop ongoing evaluation cycles • Participate in activities to adapt interventions or implementation processes as needed. • Participate in the reporting of evaluation outcomes

The ongoing development of patient engagement evaluation tools [132, 136–138] provides an opportunity to begin to consider a broader strategy for IKT research evaluation. By utilizing research approaches that have the potential to capture the complexities of purpose, process, and outcomes, such as by using hybrid designs or concurrent realist evaluation [139, 140], IKT teams can further contribute to our understanding of how dynamic partnerships between knowledge users (including patients) and researchers are formed and experienced. This in turn may give rise to more precise mechanisms to foster collaboration, enhanced decision-making, and the improved uptake of evidence-based practices in healthcare. By addressing these theoretical tensions, in addition to responding to gaps and variations within the research process, IKT researchers have the potential to further contribute to the science of patient engagement and may further expand contemporary modes of IKT practice.

## Conclusion

This paper has begun to explore some of the conceptual and theoretical considerations of patient engagement in IKT research, as well as identifying some of the potential opportunities and tensions that exist when bridging these two dominant approaches. We have argued to support the greater inclusion of patients as knowledge users in IKT as a means of increasing the relevance of research and improving health and patient outcomes. As evidence to support patient engagement and integrated pathways of research is still developing, IKT teams have a unique opportunity to contribute to both the science of IKT and patient engagement and to contribute to the development of best engagement practices. This combination of factors may further yield a greater potential for lasting change and may provide the opportunity for IKT and patient engagement to forge new ways to improve patient and health outcomes. Finally, this paper represents a call for more systematic evaluations of IKT research partnerships, particularly with respect to the ways that teams address diversity and power to foster authentic co-production and how the process contributes to the creation and application of impactful research.

## Abbreviations

CIHR: Canadian Institutes of Health Research
IKT: Integrated Knowledge Translation
KT: Knowledge Translation
NIHR: National Institute for Health Research
PCORI: Patient-Centred Outcomes Research Institute
SPOR: Strategy for Patient-Oriented Research
